# Cigarette smoke reversibly activates hypoxia-inducible factor 1 in a reactive oxygen species-dependent manner

**DOI:** 10.1038/srep34424

**Published:** 2016-09-29

**Authors:** Hiroki Daijo, Yuma Hoshino, Shinichi Kai, Kengo Suzuki, Kenichiro Nishi, Yoshiyuki Matsuo, Hiroshi Harada, Kiichi Hirota

**Affiliations:** 1 Department of Anesthesia, Kyoto University Hospital, Kyoto, Japan; 2 Department of Respiratory Medicine, Kyoto University Hospital, Kyoto, Japan; 3 Department of Anesthesiology, Kansai Medical University, Hirakata, Japan; 4 Laboratory of Cancer Cell Biology, Radiation Biology Center, Kyoto University, Kyoto, Japan; 5 Precursory Research for Embryonic Science and Technology (PRESTO), Japan Science and Technology Agency (JST), Saitama, Japan

## Abstract

Cigarette smoke (CS) is a major contributor to the development of a large number of fatal and debilitating disorders. However, the precise molecular mechanisms underlying the effects of CS in lung disease are largely unknown. To elucidate these pathophysiological processes, we examined the *in vitro* and *in vivo* effects of CS extract (CSE) and CS on the transcription factor, hypoxia-inducible factor 1 (HIF-1). CSE induced concentration- and time-dependent accumulation of HIF-1α protein in human lung epithelial-like cells under non-hypoxic conditions. Genes upregulated by HIF-1, including vascular endothelial growth factor and regulated in development and DNA damage response 1, both of which are involved in smoking-induced emphysematous changes, were increased by CSE treatment under non-hypoxic conditions *in vitro* and *in vivo*. Further investigation revealed that reactive oxygen species were generated in cells exposed to CSE and were required for CSE-mediated induction of HIF-1α protein, as was activation of phosphoinositide 3-kinase and mitogen-activated protein kinase pathways. In conclusion, we demonstrated that CSE and CS induced HIF-1 activation *in vitro* and *in vivo*, respectively. The evidence warrants further investigation to indicate that HIF-1 plays an important role in CS-induced gene expression, which is deeply involved in pulmonary cellular stress and small airway remodelling.

Cigarette smoking is a major contributing factor in the development of a large number of fatal and debilitating disorders, including degenerative diseases and cancers. Understanding the mechanisms by which smoking contributes to disease has been, and will remain, a major research focus^1^.

Cigarette smoking exacerbates several pathological events including inflammation, proteolysis, and oxidative stress, all of which may lead to the dysregulation of pulmonary cells and the development of chronic obstructive pulmonary disease (COPD)^2^,^3^. Many studies conducted on biopsies, sputum, or bronchoalveolar lavage have demonstrated the involvement of vascular endothelial growth factor (VEGF) and its receptors in vascular remodeling processes^4^,^5^. More recently, Yoshida *et al*. reported that the stress response protein, regulated in development and DNA damage response 1 (REDD1), was a major mediator of smoking-induced emphysematous changes. Expression of REDD1 is triggered by CS, resulting in increased cell death induced by oxidative stress and alveolar inflammation^6^. REDD1 is also known as Rtp801, which was identified as sharply up-regulated in C6 rat glioma cells in response to hypoxia^7^. Matrix metalloproteinases (MMPs) such as MMP2 and MMP9 are proteolytic enzymes that degrade extracellular matrix components both under physiological conditions and during pathological processes including COPD^8^,^9^. In addition, recent exploratory studies have revealed that heme oxygenase 1 (HO-1) is also involved in the development of COPD. The expression levels of VEGF, REDD1 (Rtp801), MMP-9, and HO-1 are reported to be regulated by the transcription factor, hypoxia-inducible factor 1 (HIF-1). Thus, HIF-1 may be involved in the pathogenesis of CS-induced lung pathophysiology^10^. HIF-1 is one of the most important transcription factors responsible for hypoxia-induced gene expression. HIF-1 is a heterodimer of a constitutively expressed HIF-1β subunit and an O_2_-regulated HIF-1α subunit^11^,^12^. Under normoxic conditions, members of the prolyl hydroxylase domain-containing protein/egg laying nine family hydroxylate the HIF-1α subunit on two conserved prolyl residues and an asparaginyl residue in an O_2_-, Fe^2+^-, and 2-oxoglutarate-dependent manner^13^,^14^,^15^. The hydroxylation of prolyl and asparaginyl residues in HIF-1α therefore regulates its protein stability and transactivation in an oxygen-dependent manner. Under conditions of low O_2_ and low Fe^2+^ active HIF-1α accumulates and promotes the transcription of genes involved in the adaptation to hypoxia. On the other hand, insulin-like growth factor 1 (IGF-1), human epidermal growth factor receptor 2 (HER2/neu), insulin, and the nitric oxide donor, NOC18, increase HIF-1 protein synthesis in a kinase inhibitor-sensitive manner even under non-hypoxic conditions^16^. HER2/neu activation increases the rate of HIF-1 protein synthesis via phosphoinositide 3-kinase (PI3K) and the downstream serine-threonine kinases, Akt (protein kinase B) and mammalian target of rapamycin (mTOR). mTOR phosphorylates and activates the translational regulatory proteins, eukaryotic initiation factor 4E-binding protein 1 (4E-BP1) and p70 S6 kinase^17^.

In the present study, we investigated the regulatory mechanism underlying CS-induced HIF-1 activation. We found that CSE increased reactive oxygen species (ROS) levels and stimulated HIF-1α protein translation in alveolar and bronchial epithelium-derived cells and that CS induced HIF-1-dependent gene expression in the lung tissues of mice under non-hypoxic conditions in a concentration- and time-dependent manner. These results indicate that HIF-1 may play an important role in CS exposure-induced cellular stress, inflammation, and remodeling of the alveolar and bronchial epithelium.

## Results

### CS extract (CSE) induces concentration- and time-dependent HIF-1α protein accumulation under non-hypoxic conditions

**Figure 1:**
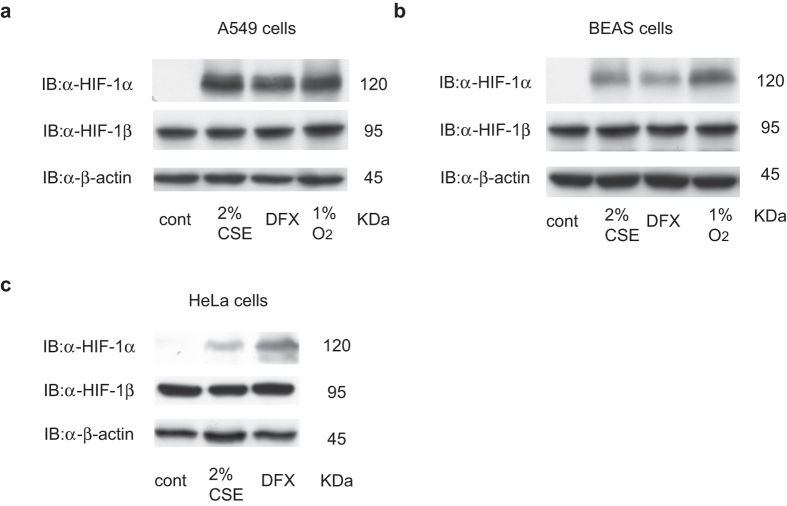
Hypoxia-inducible factor 1α (HIF-1α) is induced by cigarette smoke extract (CSE) *in vitro*. Human lung adenocarcinoma A549 cells (**a**), human bronchial epithelial BEAS-2B cells (**b**), and human cervical carcinoma HeLa cells (**c**) were exposed to the indicated concentrations of CSE in the presence of 20% O_2_, 100 μM desferrioxamine (DFX), or 1% O_2_ for 4 h. Whole-cell lysates were immunoblotted (IB) using anti-HIF-1α, HIF-1β, and β-actin antibodies.

To examine the effect of CSE on HIF-1, human lung adenocarcinoma A549 (Fig. 1a and Supplementary 1 and 2a) and human bronchial epithelial BEAS-2B cells (Fig. 1b and Supplementary 2b) were exposed to 2% CSE under non-hypoxic (20% O_2_), 100 μM desferrioxamine (DFX) or hypoxic (1% O_2_) conditions for 4 h. In both cell lines, CSE promoted the accumulation of HIF-1α to a similar extent as DFX or exposure to 1% O_2_^18^. Expression of HIF-1β or β-actin was not affected by CSE treatment. CSE also induced HIF-1α protein accumulation in the cervical carcinoma HeLa cell line (Fig. 1c and Supplementary 2c).

**Figure 2:**
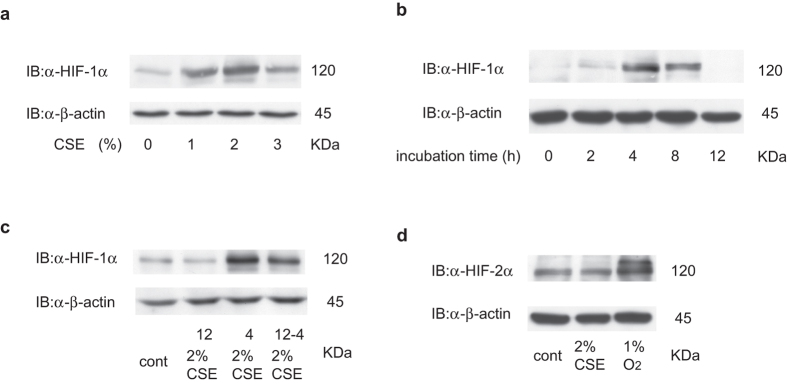
Cigarette smoke extract (CSE) induces concentration- and time-dependent accumulation of hypoxia-inducible factor 1α (HIF-1α) protein under non-hypoxic conditions. Human lung adenocarcinoma A549 cells were exposed to the indicated concentrations of CSE under 20% O_2_ (**a,c**) and 1% O_2_ (**d**) for 4 h or to 2% CSE for the indicated time-periods (**b**). The cell lysates were immunoblotted (IB) with the indicated antibodies. Experiments were repeated at least twice in triplicate and representative blots are shown.

Next, we investigated the concentration-dependency of the CSE effect on HIF-1α protein expression in A549 cells. A549 cells were exposed to 1, 2, or 3% CSE under 20% O_2_ for 4 h. Two percent CSE induced greater accumulation of HIF-1α protein than 1% CSE, while 3% CSE produced an inhibitory effect (Fig. 2a and Supplementary 2d). The effect of CSE peaked at 4 h and HIF-1α protein levels then gradually declined, returning to baseline levels by 12 h (Fig. 2b and Supplementary 2e). To examine the mechanisms behind this transient CSE effect in A549 cells, A549 were exposed to 2% CSE for 12 h and then exposed to 2% CSE again for 4 h. The cell extracts were then immunoblotted using an anti-HIF-1α antibody. HIF-1α protein levels were elevated in cells exposed to 2% CSE, even at 12 h (Fig. 2c). We also studied HIF-2α protein accumulation in A549 cells. In contrast to HIF-1α, CSE treatment did not induce HIF-2α protein accumulation (Fig. 2d).

CS is known to have various negative effects on alveolar epithelial cells. We therefore investigated cell viability and apoptosis in CSE-exposed A549 cells. Incubation with 1 or 2% CSE for 12 h did not affect cell viability (Supplementary 3a). In addition, cleavage of poly (ADP-ribose) polymerase was not detected in the lysates of CSE-treated A549 cells (Supplementary 3b). Overall, these data showed that the concentration of CSE used in the present study was not toxic to A549 cells.

### CSE effects on gene expression were HIF-1-dependent

**Figure 3:**
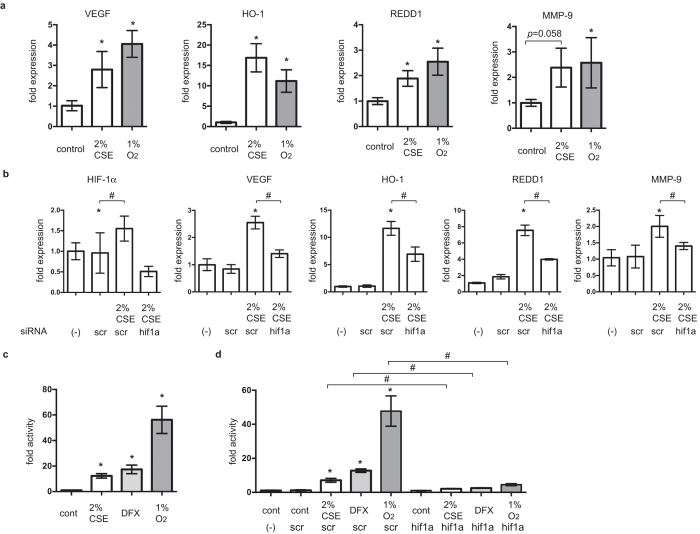
Effect of cigarette smoke extract (CSE) on the expression of hypoxia-inducible factor 1 (HIF-1)-dependent genes. (**a**) A549 cells were cultured for 6 h with or without 2% CSE under 20% O_2_ or 1% O_2_ prior to analysis of vascular endothelial growth factor (VEGF), heme oxygenase-1 (HO-1), regulated in development and DNA damage response 1 (REDD1), and matrix metalloproteinase 9 (MMP-9) mRNA levels using real-time reverse transcriptase polymerase chain reaction (RT-PCR). Fold expression was calculated relative to untreated control cells. (**b**) A549 cells were transfected with small interfering RNA (siRNA) targeting HIF-1α (hif1a) or a negative control (scr) and exposed to CSE or 1% O_2_ for 6 h. HIF-1α, VEGF, and REDD1 mRNAs were analyzed by RT-PCR and fold expression was calculated relative to untreated cells. (**c,d**) A549/5HRE-Luc cells (**c**) or A549/5HRE-Luc cells transfected with hif1a or scr siRNAs (**d**) were exposed to the indicated conditions for 8 h prior to analysis of luciferase activity. Fold activity was calculated relative to untreated cells and experiments were repeated at least three times in triplicate. Data are presented as the mean ± SD; *p < 0.05, as compared with control (no treatment); ^#^*p* < 0.05 for the indicated comparisons.

We investigated the effects of CSE on HIF-1-mediated gene expression in A549 cells. mRNA expression was assayed using semi-quantitative real-time reverse transcriptase polymerase chain reaction (RT-PCR). Exposure to 2% CSE induced the mRNA expression of VEGF, HO-1, REDD1, and MMP-9 within 8 h in the presence of 20% O_2_; this was comparable to the effects of exposure to 1% O_2_ (Fig. 3a). Expression of mRNAs encoding glucose transporter 1, lactate dehydrogenase A (LDHA), and BCL2/adenovirus E1B 19 kd-interacting protein were also investigated (Supplementary 4). To investigate the role of HIF-1α in the induction of VEGF and REDD1 mRNA expression, A549 cells were exposed to small interfering RNA (siRNA) targeting HIF-1α. The resulting knockdown of HIF-1α decreased CSE-mediated induction of VEGF, HO-1, REDD1, and MMP-9 mRNAs (Fig. 3b). Next, we investigated the effect of CSE on HIF-1 activity in A549 cells using a hypoxia-responsive element (HRE)-luciferase reporter construct^19^,^20^. Exposure to 2% CSE, 100 μM DFX, or 1% O_2_ promoted HRE-dependent gene expression (Fig. 3c). siRNA-mediated knockdown of HIF-1α mRNA decreased the CSE-induced reporter gene expression (Fig. 3d). Together, these study findings indicate that HIF-1α was exclusively responsible for CSE-mediated induction of VEGF, HO-1, REDD1, and MMP-9 gene expression.

### CSE does not prolong the half-life of HIF-1α protein

**Figure 4:**
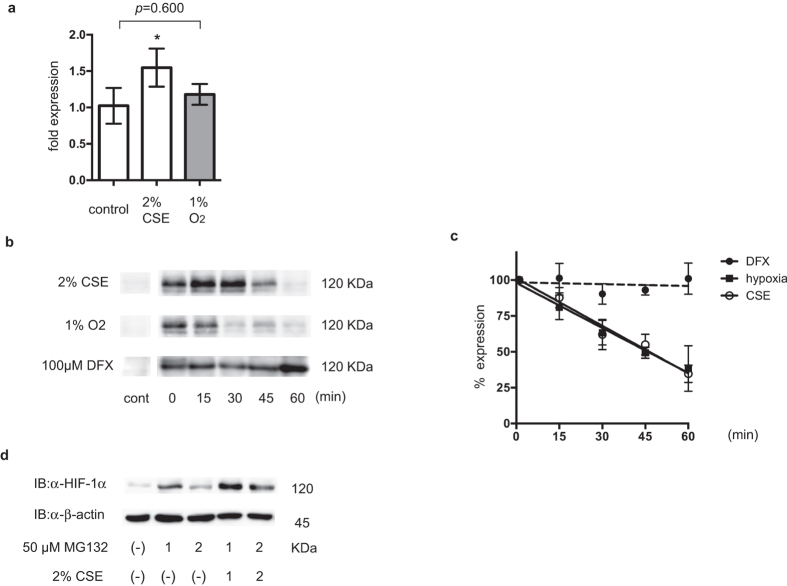
Cigarette smoke extract (CSE) effects on hypoxia-inducible factor 1α (HIF-1α) protein stability and synthesis. (**a**) A549 cells were exposed to the indicated conditions for 4 h prior to analysis of HIF-1α mRNA using real-time reverse transcriptase polymerase chain reaction (RT-PCR). Fold expression was calculated relative to untreated cells. Data are presented as the mean ± SD; *p < 0.05, as compared with the control (no treatment); ^#^*p* < 0.05 for the indicated comparisons. (**b,c**) A549 cells were exposed to the indicated conditions for 4 h and then cycloheximide (CHX; 100 μM). The cells were incubated for the indicated times prior to whole cell lysate immunoblotting using anti-HIF-1α and anti-β-actin antibodies (**b**). Mean expression ratios, relative to control cells, were calculated from densitometry of two independent immunoblots and shown in (**c**). (**d**) A549 cells were treated as indicated under 20% O_2_ prior to immunoblotting (IB) for the indicated proteins.

Treatment with 2% CSE induced HIF-1α mRNA expression, as determined using semi-quantitative RT-PCR. In contrast, 1% O_2_ did not affect HIF-1α mRNA expression (Fig. 4a). To determine whether CSE treatment affected the HIF-1α protein half-life, A549 cells were exposed to 2% CSE, 100 μM DFX, or 1% O_2_ for 4 h to induce HIF-1α protein accumulation, and then the protein synthesis inhibitor, cycloheximide (CHX), was added (Fig. 4). In the presence of CHX, the half-life of HIF-1α was over 60 min in DFX-treated cells but around 45 min in CSE-treated cells (Fig. 4b,c). For further investigation of the stability of HIF-1α protein, cells were treated with or without CSE along with the proteasome inhibitor, MG132. Cells exposed to CSE had higher levels of HIF-1α protein than those that were not exposed to CSE (Fig. 4d). These results indicated that HIF-1α protein expression in CSE-treated cells required ongoing protein synthesis. These experiments suggest that CSE-stimulated HIF-1α protein expression is due, at least in part, to increased HIF-1α mRNA levels and synthesis of HIF-1α protein.

### Impact of kinase inhibitors on CSE-induced HIF-1 activation

**Figure 5:**
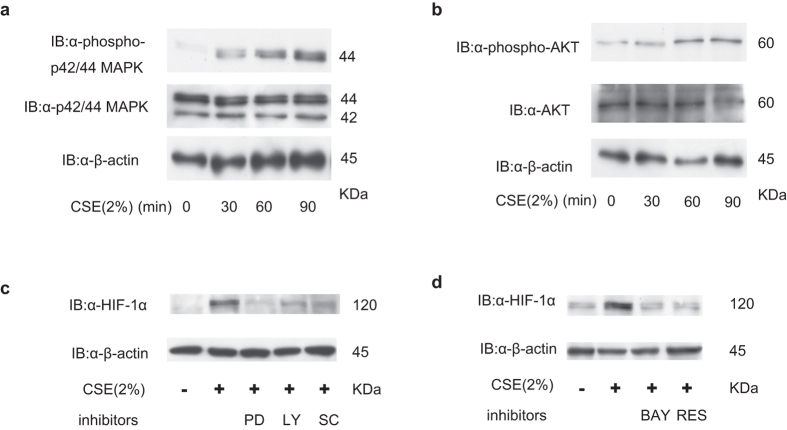
Impact of kinase inhibitors on cigarette smoke extract (CSE)-induced hypoxia-inducible factor 1 (HIF-1) activation. A549 cells were exposed to 2% CSE for the indicated time-periods (**a,b**) prior to immunoblotting (IB) whole cell lysates for phosphorylated and total-p42/44 mitogen-activated protein kinase (MAPK) (**a**) or Akt (**b**) and β-actin. (**c**) A549 cells were incubated with or without 2% CSE for 4 h in the presence of 20-μM PD98059 (PD), 25-μM LY294002 (LY), or 100-μM SC-514 (SC) under 20% O_2_ prior to IB of whole cell lysates, as indicated. (**d**) A549 cells were exposed to 2% CSE with or without the NF-κB inhibitors, 20-μM BAY11-7082 (BAY) or and 20-μM resveratrol (RES) for 4 h prior to IB of whole cell lysates, as indicated.

CSE has been reported to strongly activate PI3K and mitogen-activated protein kinases (MAPK)^21^,^22^; several reports indicate the involvement of PI3K and MAPK signaling pathways in HIF-1 activation^23^. To investigate whether CSE treatment affected PI3K and MAPK activation in our *in vitro* model, we examined the phosphorylation of p42/44 MAPK and Akt using immunoblotting. Phosphorylation of p42/44 MAPK (Fig. 5a) and Akt (Fig. 5b) was induced by 30-min CSE treatment. The kinase inhibitors, LY294002, PD98059, and SC-514, inhibited HIF-1α protein accumulation in CSE-treated A549 cells (Fig. 5c). Next, to examine the involvement of the transcription factor, nuclear factor-κB (NF-κB), in CSE-induced HIF-1α protein accumulation, A549 cells were exposed to 2% CSE in the presence or absence of the NF-κB inhibitors, BAY11-7082 (20 μM) or resveratrol (20 μM) for 4 h. Treatment with BAY11-7082 or resveratrol suppressed HIF-1α protein expression (Fig. 5d).

### Critical involvement of ROS in HIF-1 activation

**Figure 6:**
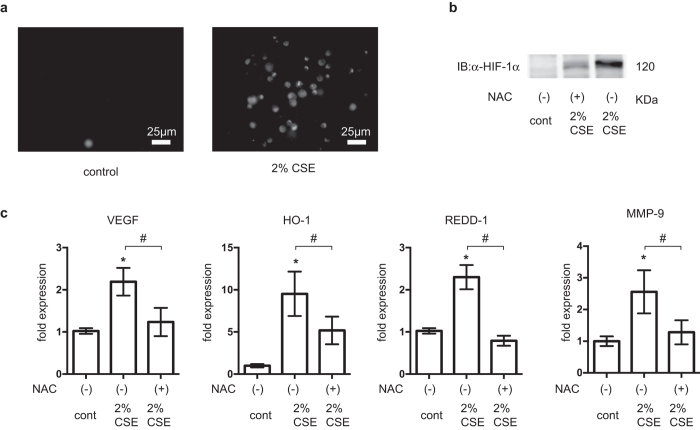
Involvement of reactive oxygen species (ROS) in hypoxia-inducible factor 1 (HIF-1) activation. (**a**) ROS generation in A549 cells was monitored by loading with 2′,7′-dichlorodihydrofluorescin diacetate at 37 °C for 20 min prior to 2% CSE exposure for 4 h. A549 cells were incubated with or without 2% CSE and 10 mM N-acetylcysteine (NAC) under 20% O_2_ for 4 h prior to (**b**) whole-cell lysate immunoblotting (IB) for HIF-1α and (**c**) determination of the mRNA levels of vascular endothelial growth factor (VEGF), heme oxygenase-1 (HO-1), and regulated in development and DNA damage response 1 (REDD1) using real-time reverse transcriptase polymerase chain reaction. Data are presented as the mean ± SD; *p < 0.05, as compared with control (no treatment); ^#^*p* < 0.05 for the indicated comparisons.

CSE also increased the levels of ROS (Fig. 6a). To investigate the role of ROS, we studied the effect of a potent antioxidant, N-acetylcysteine (NAC), on CSE-induced HIF-1 activation. NAC strongly suppressed HIF-1α protein accumulation in CSE-treated A549 cells (Fig. 6b). NAC also suppressed CSE-induced mRNA expression of VEGF, HO-1, REDD1, and MMP-9 (Fig. 6c).

### Effect of major components of CSE on HIF-1α accumulation

**Figure 7:**
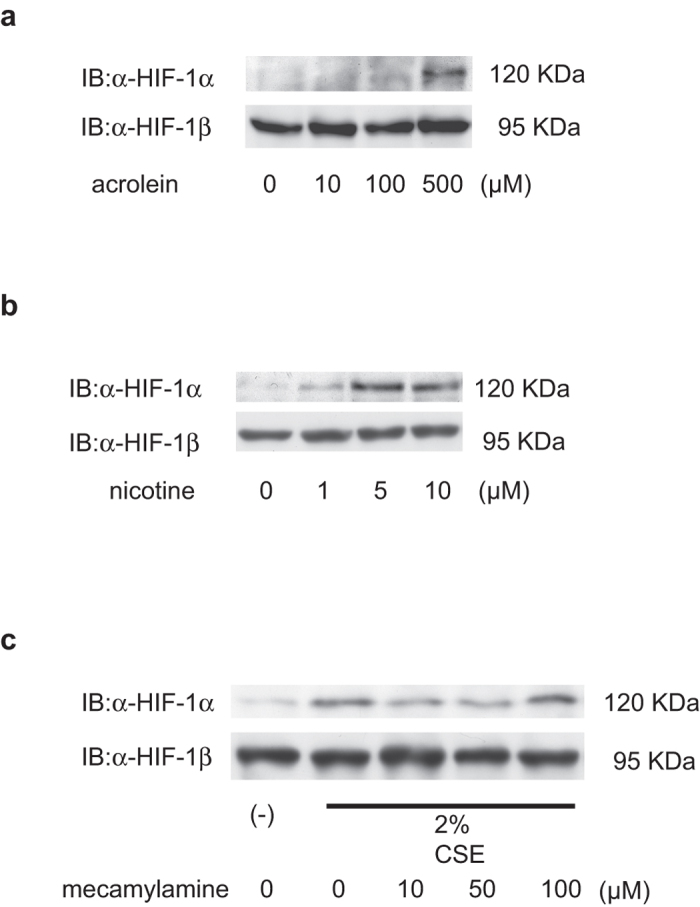
Effect of acrolein and nicotine on hypoxia-inducible factor 1 (HIF-1) protein expression. A549 cells were exposed to the indicated concentrations of (**a**) acrolein, (**b**) nicotine, and (**c**) mecamylamine with or without 2% CSE under 20% O_2_ prior to immunoblotting (IB) analysis of whole-cell lysates for HIF-1α and HIF-1β.

CS is a mixture of over 4,000 chemicals and the relative lung toxicity effects of these remain unclear. We investigated the effects of an aldehyde (acrolein) and nicotine, which are major components of CSE^24^,^25^, on HIF-1 activation in A549 cells. Acrolein (500 μM) and nicotine (5 μM) induced accumulation of HIF-1α protein (Fig. 7a,b). The HIF-1α protein accumulation induced by nicotine treatment was not inhibited by the nicotine antagonist, mecamylamine^25^ (Fig. 7c). These results indicate that aldehydes and nicotine are not the only CSE constituents involved in CSE-induced HIF-1α protein accumulation.

### CS exposure induces HIF-1 activation *in vivo*

**Figure 8:**
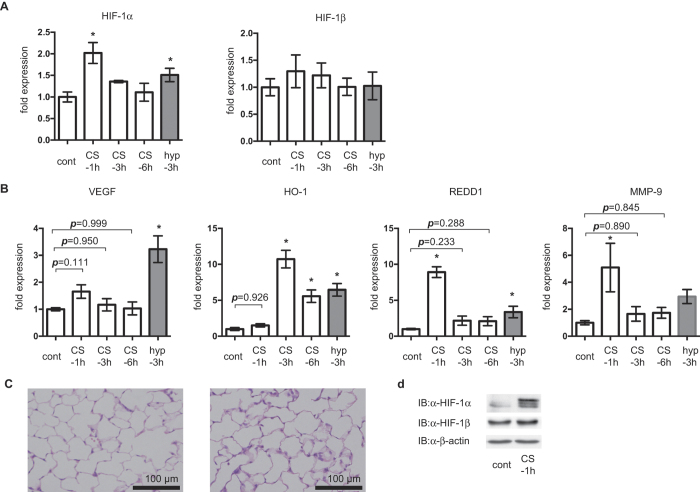
Hypoxia-inducible factor 1 (HIF-1) is activated in the lungs of mice exposed to cigarette smoke (CS). Mice were exposed to air (cont), CS from 10 filtered cigarettes (CS) or 1% O_2_ (hyp) for 50 min followed by air for 1 h (CS-1 h), 3 h (CS-3 h and hyp-3 h), 4 h (cont), or 6 h (CS-6 h) prior to analyzing lung (**a**) HIF-1α and HIF-1β mRNA levels, and (**b**) vascular endothelial factor (VEGF), heme oxgenase-1 (HO-1), regulated in development and DNA damage response 1 (REDD1), and matrix metalloproteinase 9 (MMP-9) mRNA levels using real-time reverse transcriptase polymerase chain reaction. Data are presented as the mean ± SD of three independent animals; *p < 0.05, as compared with the control (20% and no treatment), ^#^*p* < 0.05 for the indicated comparisons. (**c**) Immunohistochemical staining for HIF-1α in mouse lung tissue. Mice were exposed to air (control) or CS for 1 h. Figures are representative of 2 tissues sections from 2 mice. Scale bars: 100 μm. (**d**) Western blot analysis for HIF-1 proteins in in mouse lung tissue. Mice were exposed to air (control) or CS for 1 h then harvested for protein extrication followed by Western blot analysis using anti-HIF-1α, HIF-1β and β-actin antibodies.

Expression of HIF-1α mRNA, but not HIF-1β mRNA, was induced in mice exposed to CS (Fig. 8a); this was consistent with the *in vitro* study of A549 cells. Acute exposure to CS increased lung VEGF, LDHA, HO-1, REDD1, and MMP-9 mRNA expression (Fig. 8b) to a similar extent as was observed following exposure to 1% O_2_. Expression of GLUT1 mRNA was also examined (Supplementary 5). The kinetics of these changes in mRNA levels were also analyzed. VEGF and REDD-1 expression peaked at 1 h, while expression of GLUT1 and HO-1 mRNAs peaked at 3 h. By 6 h post-exposure, mRNA expression had decreased. Finally, we assayed the expression of HIF-1α protein at 1h of CS exposure, because mRNA expression of HIF-1 and its downstream genes peaked at 1h rather than 3h or 6h. The immunohistochemical study indicated that positive HIF-1α immunostaining was observed globally in the lung alveolar tissue after exposure to CS (Fig. 8c). In addition, Western blot analysis also indicated increase of HIF-1α protein in lung tissue after exposure to CS (Fig. 8d).

## Discussion

The present study has provided a novel insight into the regulatory mechanisms underlying CS-induced HIF-1 activation. CSE treatment induced HIF-1α protein accumulation, as well as altering the expression of a series of downstream genes in cells derived from the alveolar and bronchial epithelium; *in vivo* CS exposure also increased protein and mRNA levels of the HIF-1α subunit and downstream genes. Intriguingly, we also found that the activation of HIF-1 was reversible. The effect of CSE on HIF-1α expression peaked at 4 h and then gradually declined, returning to baseline levels within 12 h in A549 cells. The cells can response to CSE even after 12 h treatment to increase the HIF-1α protein expression. This was essentially consistent with the *in vivo* results shown in Fig. 8. Expression of HIF-1α protein peaked within 1 h of CS exposure and declined to the baseline level within 6 h. These findings indicated that chain smokers are likely to induce continuous HIF-1 protein expression and HIF-1 activation in the bronchial and alveolar lung epithelium, resulting in sustained mRNA expression of genes that are downstream of HIF-1. Moreover, we also provided experimental evidence that ROS and the activation of several kinases play an essential role in the induction of HIF-1α mRNA expression and facilitate HIF-1α protein translation.

The steady-state HIF-1α protein levels are determined by regulation of its stability and synthesis. HIF-1α stability is regulated by HIF-1α prolyl hydroxylases in an O_2_-dependent manner^26^. O_2_ is one of the substrates involved in the HIF-α–hydroxylase reaction and hypoxia decreases hydroxylated HIF-α protein levels and increases the half-life of HIF-1α, resulting in HIF-1 activation. However, CSE did not prolong the half-life of HIF-1α protein in the present study (Fig. 4). Our investigation of the mechanisms underlying HIF-1 activation revealed that CSE activated the PI3K/Akt and MAPK signaling pathways, thereby increasing expression of HIF-1α mRNA and the synthesis and transactivation activity of HIF-1α protein, without affecting its stability (Fig. 3). This result was consistent with earlier publications showing that activation of the PI3K/Akt/mTOR and MAPK pathways increased the rate of HIF-1α mRNA expression and protein synthesis^17^,^20^,^27^,^28^. Physiological stimuli other than hypoxia can also induce HIF-1 activation and the subsequent transcription of hypoxia-inducible genes. Signaling via the HER2/neu or IGF-1 receptor tyrosine kinase induces HIF-1 expression in an O_2_-independent manner. HER2/neu activation increases the rate of HIF-1α protein synthesis via PI3K and the downstream serine/threonine kinases, Akt and mTOR^23^,^29^. Other stimuli that activate HIF-1, such as IGF-1, insulin, and the nitric oxide donor, NOC18, can increase HIF-1α protein synthesis in a kinase inhibitor-sensitive manner^20^. As shown in Fig. 5C, induction of HIF-1α protein expression by CSE was blocked by inhibition of PI3K or MAPK. CSE induces phosphorylation of Akt and p42/44 MAPK, both of which are involved in the control of protein translation (Fig. 5a,b). Taken together, these data indicate that CSE increases the rate of HIF-1α protein synthesis by increasing HIF-1α mRNA levels and by activating the PI3K/Akt and MAPK pathways.

Zhou *et al*. reported that tumor necrosis factor-α activates the NF-κB, PI3K, and MAPK signaling pathways that lead to Bcl-2 expression, which in turn induces Internal Rebosomal Entry Site (IRES)-dependent HIF-1 mRNA translation and HIF-1 protein synthesis in LLC-PK1 cells^30^. This evidence prompted us to investigate the involvement of NF-κB in CSE-induced HIF-1α accumulation. Two types of NF-κB inhibitors suppressed CSE-induced HIF-1α protein accumulation in A549 cells. This was not consistent with our previous report that lipopolysaccharide-induced HIF-1 activation was not dependent on NF-κB in the human monocyte THP-1 cell line. Taken together, the evidence indicates that induction of HIF-1 expression is stimulus and/or cell-type specific, and that ROS is involved as an essential intermediate.

Another novel insight of the present study is the finding that HIF-1 activation was ROS-dependent. CS is known to induce oxidative stress and inflammation in pulmonary tissues and cells, both *in vitro* and *in vivo*. There are many reports indicating that CSE induces ROS production and oxidative stress^31^,^32^,^33^. In fact, ROS were generated in A549 cells exposed to CSE under the present experimental conditions (Fig. 6a). Elevated ROS have been implicated in HIF-1 signaling^25^,^34^,^35^, and studies have shown that mitochondria-derived ROS are both necessary and sufficient to stabilize and activate HIF-1^36^,^37^,^38^,^39^. Thus, mitochondria-derived ROS are involved in the regulation of HIF-1α protein stability. However, the present study demonstrated that CSE-derived ROS did not affect the HIF-1α protein half-life. This suggested that different molecular mechanisms were involved in the CS- and hypoxia-mediated inductions of HIF-1α protein expression. The antioxidant, NAC, almost completely suppressed CSE-induced HIF-1 activation (Fig. 6b) and CSE treatment promoted ROS generation, suggesting that this CS-mediated effect required ROS generation. ROS-MAPK/Akt signaling plays an essential role in CS-induced HIF-1 activation. This is similar to the situation previously reported in relation to lipopolysaccharide-induced HIF-1 activation in macrophage-like cells, and ROS signaling^40^.

CS contains over 4,000 chemical constituents, including high concentrations of oxidants. The present study tested the effects of two representative constituents, acrolein and nicotine (Fig. 7). Although acrolein and nicotine induced HIF-1α protein accumulation in a concentration-dependent manner, the extent of the increase was lower than that observed following CSE treatment (Fig. 7). Furthermore, a nicotine receptor antagonist (mecamylamine) did not block the effect of CES on HIF-1α protein levels in A549 cells.

This study has several limitations. The upregulation of VEGF observed in the present study is consistent with these previous reports and further investigations using immunohistochemistry or *in situ* hybridization are needed to confirm the localization of this additional VEGF^10^. Expression of HO-1, REDD-1, and MMP-9 was exclusively investigated by semi-quantitative RT-PCR. However, the expression of the proteins were not investigated by either Western blot or immunohistochemisty *in vivo* settings. Finally, we did not investigate the involvement of HIF-1 in CS-induced COPD *in vivo* models.

There are some other reports indicating that HIF-1 activity is affected by CS exposure. Michaud *et al*. reported that CS exposure impaired angiogenesis by inhibiting VEGF, due to the decreased expression of HIF-1α under hypoxic conditions^41^. In contrast, Zhang *et al*. demonstrated that nicotine, a major constituent of CS, stimulated HIF-1α protein accumulation and VEGF expression in human non-small cell lung cancer^42^. In addition, Sun. *et al*. showed that chronic tobacco exposure promoted normoxic HIF-1α activation in squamous cell carcinoma^43^. These contrasting results might reflect the different cell types studied or variations in the CSE treatment protocols employed.

The findings of the present study demonstrated that CS exposure stimulated HIF-1α protein synthesis and induced HIF-1 activation both *in vitro* under 20% O_2_ conditions and *in vivo* under ambient air conditions. CSE induced the expression of a series of COPD-related genes such as REDD1 and VEGF via HIF-1 activation in a ROS-dependent manner and this may result in apoptosis, excessive protease production, and lung inflammation.

## Methods

### Cell culture and reagents

A549 and BEAS-2B cells were maintained in Dulbecco’s modified Eagle’s medium (DMEM) and HeLa cells were maintained in RPMI 1640 medium supplemented with 10% fetal bovine serum, 100 U/mL penicillin, and 0.1 mg/mL streptomycin. DFX, LY294002, PD98059, and the anti-β-actin antibody were obtained from Sigma (St. Louis, MO). CHX, the cell-permeable proteasome inhibitor, Z-Leu-Leu-Leu-aldehyde (MG132), NAC, and dithiothreitol (DTT) were obtained from Calbiochem (San Diego, CA). A mouse anti-HIF-1α antibody was purchased from BD Biosciences (San Jose, CA) and a goat polyclonal anti-HIF-1α antibody was purchased from R&D Systems (Minneapolis, MN).

### Preparation of CSE

CSE was prepared using a modification of a previously published method^24^. Briefly, 5 filtered cigarettes were smoked consecutively through an experimental apparatus with a constant airflow (0.3 L/min) driven by an air compressor^24^; each cigarette contained 1.3 mg nicotine and 15 mg tar, according to the manufacturer’s report. The smoke was bubbled through 10 mL DMEM, supplemented with 20 mM HEPES. The CSE obtained was then filtered through a 0.22-μm filter (Millipore, Bedford, MA). The CSE was prepared immediately before each experiment, unless stated otherwise.

### Immunoblot assays

Whole cell lysates were prepared using ice-cold lysis buffer containing 0.1% sodium dodecyl sulfate (SDS), 1% Nonidet P-40, 5 mM ethylenediaminetetraacetic acid, 150 mM NaCl, 50 mM Tris-Cl (pH 8.0), 2 mM DTT, 1 mM sodium orthovanadate, and Complete Protease Inhibitor™ (Roche Diagnostic, Tokyo, Japan), as described previously^44^,^45^. Samples were centrifuged at 10,000 × *g* to pellet cell debris. For HIF-1α and HIF-1β analyses, 100 μg of protein was fractionated by SDS-polyacrylamide gel electrophoresis (SDS-PAGE; 7.5% gel) and subjected to an immunoblot assay using the indicated primary antibodies at a dilution of 1:1000. Anti-β-actin mouse monoclonal antibody (Sigma) was used at a dilution of 1:5000 as the control. Horseradish peroxidase-conjugated sheep anti-mouse IgG (GE Healthcare, Piscataway, NJ) was used as the secondary antibody, at a dilution of 1:1000. The signal was developed using enhanced chemiluminescence reagent (GE Healthcare). The intensity of each band was quantified using Image J software^44^.

### Gene silencing by siRNA

A549 cells were grown until 30–50% confluence prior to plating on a 24-well plate using DMEM without antibiotics. The cells were then transfected with the Validated Stealth RNAi (100 pmol/mL) for HIF-1α (5′-GGAUGCUGGUGAUUUGGAUAUUGAA′) or with the Stealth RNAi Negative Control Kit (both from Invitrogen Corp., Carlsbad, CA) using Lipofectamine RNAiMAX (Invitrogen Corp.), according to the manufacturer’s instructions^46^. Transfected cells were incubated in a normoxic incubator for 24 h following CSE treatment.

### Semi-quantitative RT-PCR analysis

Total RNA was extracted from A549 cells using the TaKaRa FastPure RNA kit (Takara Bio, Ohtsu, Japan), according to the manufacturer’s instructions. First-strand synthesis and RT-PCR were performed using the One-step SYBR PrimeScript RT-PCR kit (TAKARA, Ohtsu, Japan), according to the manufacturer’s protocol. Amplification and detection were performed using the Applied Biosystems 7300 Real-time PCR System (Applied Biosystems, Foster City, CA). PCR primers were purchased from Qiagen. The change in expression of each target mRNA was calculated relative to the level of 18S rRNA^47^,^48^.

### Reporter gene assay

A549/5HRE-Luc cells, which express the luciferase gene under the control of the 5HRE promoter, were described previously^49^,^50^. Cells were seeded in 24-well plates (5 × 10^4^ cells/well) and subjected to the indicated treatments. The cells were washed with phosphate-buffered saline and lysed with 100 μM Passive Lysis Buffer (Promega, Madison, WI). The luciferase assay was performed using Luciferase Assay Reagent (Promega), according to the manufacturer’s instructions.

### Animal studies

Three-month-old male C57BL6/J mice were purchased from Japan SLC (Shizuoka, Japan). Food and water were available *ad libitum* and the mice were maintained under controlled environmental conditions (24 °C, 12-h light/dark cycles)^48^. Mice were divided into 5 study groups: control (maintained in the air for 4 h), CS–1 h (exposed to CS for 50 min and maintained in the air for 1 h), CS–3 h (exposed to CS for 50 min and maintained in the air for 3 h), CS–6h (exposed to CS for 50 min and maintained in the air for 6 h), and hypoxia–3 h (exposed to 1% O_2_ for 50 min and maintained in the air for 3 h). The animal protocols were approved by the Animal Research Committee of Kyoto University (med-kyt #12156, Kyoto University, Japan) and all experiments were performed in accordance with the National Institute of Health Guidelines for the Care and Use of Laboratory Animals. Blood pressure, heart rate, and peripheral O_2_ saturation were measured by a tail-cuff sphygmomanometer (model MK-1030; Muromachi Kikai, Tokyo, Japan) and a MouseOx pulse oximeter (Starr Life Sciences, Oakmont, PA) during the experiments. At the end of the experiments, the mice were killed by cervical dislocation. The lungs were rapidly removed, frozen in liquid nitrogen, and stored at −80 °C.

### Short-term CS exposure

University of Kentucky research grade cigarettes (code 2R4F) were used in this *in vivo* study^51^. Mice were placed in polycarbonate chambers and exposed to CS generated from 10 filter-cut standard cigarettes for 15 min, using SG-200 smoke generator equipment (Shibata Scientific Technology Ltd., Tokyo, Japan)^52^,^53^.

### Cytotoxicity and cellular proliferation assays

Cellular proliferation and its inhibition were determined by the CellTiter 96 Aqueous Non-Radioactive Cell Proliferation Assay™ (Promega). Upon completion of a given experiment, 333 μg/mL MTS (3-(4,5-dimethylthiazol-2-yl)-5-(3-carboxymethoxyphenyl)-2-(4-sulfophenyl)-2H-tetrazolium) and 25 μM phenazine methosulfate were added to each well of the 96-well plate for 1 h at 37 °C. This allowed the dehydrogenases in metabolically active cells to reduce the MTS. The soluble MTS formazan product was measured at 490 nm in a microplate reader (M-Tmax; Wako Ind., Ltd.). The optical density (OD) was directly proportional to the number of living cells. Cytotoxicity (%) was calculated using: [(OD of control cells − OD of treated cells)/OD of control cells] × 100.

### Live cell ROS imaging

Intracellular ROS generation in CSE-treated A549 cells was analyzed by a live cell time-lapse imaging system (BioStation IM; Nikon, Tokyo) using a ROS-sensitive dye, 2′,7′-dichlorodihydrofluorescin diacetate, at 37 °C and 5% CO_2_; phase-contrast and fluorescent images were acquired every 15 min^54^,^55^.

### VEGF ELISA

A549 cells were grown in 12-well plates and treated as indicated. The cell culture supernatants were collected and VEGF was quantified using the Human VEGF Quantikine ELISA kit™ (R&D Systems, Minneapolis, MN) in accordance with the manufacturer’s instructions. Quantification was performed by determining the absorbance at 450 nm using a microplate reader (M-Tmax; Wako Ind., Ltd.), with 570 nm as the reference wavelength.

### Immunohistochemistry

Immunohistochemistry was performed as described previously^56^,^57^. Lung tissue section on glass slides were washed 6 times (5 min each) in phosphate-buffered saline (PBS) and incubated with 1% normal goat serum in PBS for 30 min. Subsequently, rabbit polyclonal anti-HIF-1α (AB 1536; R&D Systems) diluted 1:200 was applied overnight at 4 °C. The sections were then incubated with biotinylated goat anti-rabbit serum (secondary antibody) diluted 1:300 in PBS for 40 min, followed by 6 washes in PBS (5 min each). Avidin-biotin-peroxidase complex (ABC-Elite, Vector Laboratories, Burlingame, CA) was then applied for 50 min at a dilution of 1:100 in BSA. After washing 6 times in PBS (5 min each) the signal was visualized using diaminobenzidine, and the nuclei were counterstained with hematoxylin.

### Statistical analysis

All experiments were repeated on at least two occasions in triplicate. Data were expressed as the mean ± standard deviation (SD) and analyzed by one-way analysis of variance, followed by Turkey’s multiple comparisons test. All statistical analyses were performed with EZR (Saitama Medical Center, Jichi Medical University), which is a graphical user interface for R (The R Foundation for Statistical Computing, version 3.2.2)^58^. More precisely, it is a modified version of R commander (version 2.2–2) and includes statistical functions that are frequently used in biostatistics. A *p*-value of <0.05 was considered statistically significant.

## Additional Information

**How to cite this article**: Daijo, H. *et al*. Cigarette smoke reversibly activates hypoxia-inducible factor 1 in a reactive oxygen species-dependent manner. *Sci. Rep.* **6**, 34424; doi: 10.1038/srep34424 (2016).

## Supplementary Material

Supplementary Information
