# Supplementary material for: Cigarette smoke reversibly activates hypoxia-inducible factor 1 in a reactive oxygen species-dependent manner
Source: Sci Rep. 2016 Sep 29;6:34424. doi: 10.1038/srep34424 (PMC5041075; doi:10.1038/srep34424)
Supplement: Supplementary Information [file srep34424-s1.pdf]

Supplementary Information

Cigarette smoke reversibly activates hypoxia-inducible factor 1 in a reactive oxygen species-dependent manner

Authors:

Hiroki Daijo [1], Yuma Hoshino [2], Shinichi Kai [3], Kengo Suzuki [3], Kenichiro Nishi [3], Yoshiyuki Matsuo [3], Hiroshi Harada [4, 5, 6], and Kiichi Hirota [3 *]

Affiliations:

[1] Department of Anesthesia, Kyoto University Hospital, Kyoto, Japan; [2] Department of Respiratory Medicine, Kyoto University Hospital, Kyoto, Japan; [3] Department of Anesthesiology, Kansai Medical University, Hirakata, Japan; [4] Department of Radiation Oncology and Image-applied Therapy, Kyoto University Graduate School of Medicine, Kyoto, Japan; [5] Group of Radiation and Tumor Biology, Career-Path Promotion Unit for Young Life Scientists, Kyoto University, Kyoto, Japan; [6] Precursory Research for Embryonic Science and Technology (PRESTO), Japan Science and Technology Agency (JST), Saitama, Japan

Supplementary Figure S1

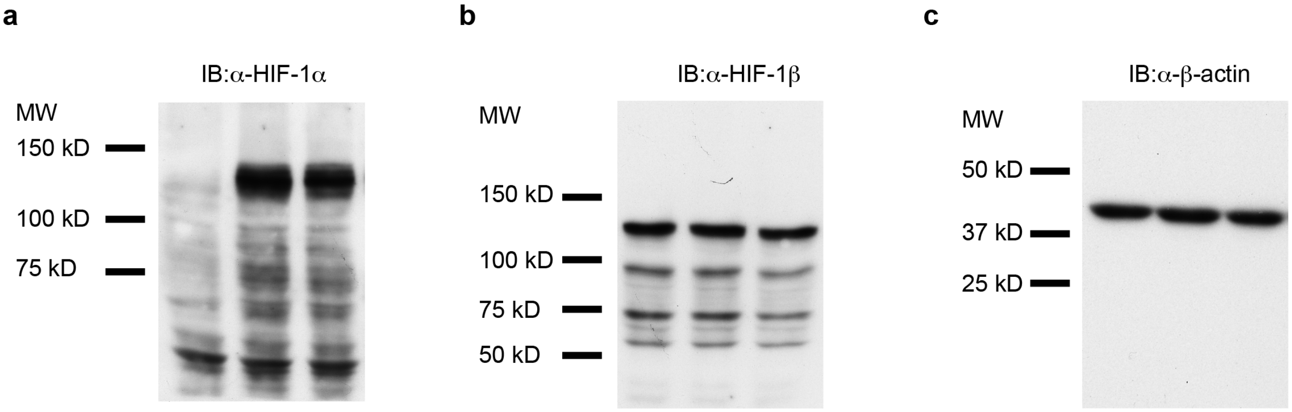

Supplementary Figure S1 | Full blot of a typical western blot

A typical Western blot for the figure 1a was demonstrated with label for the molecular weight marker.

Supplementary Figure S2

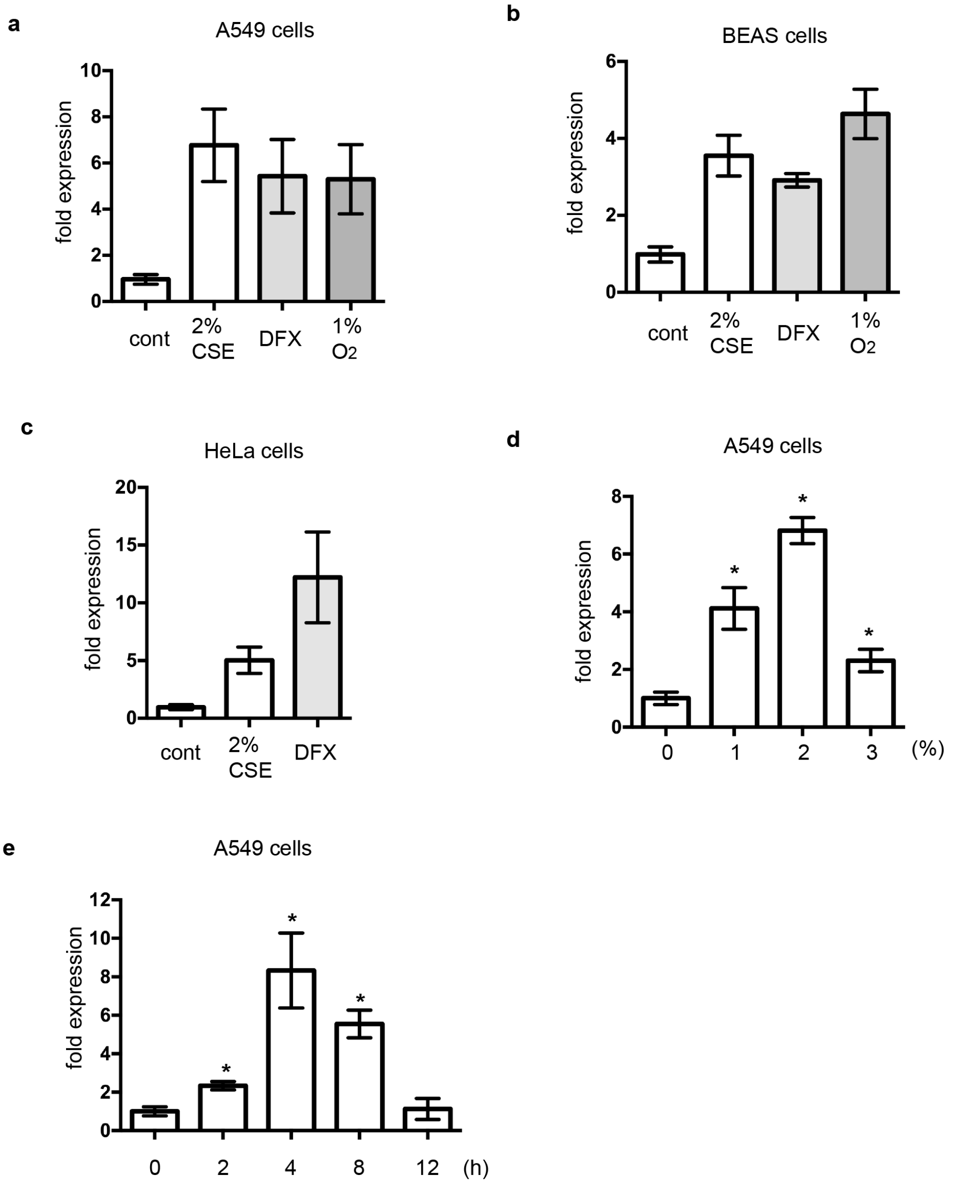

Supplementary Figure S2 | Results of densitometric analysis

The results of densitometric analysises were demonstrated. (a), (b) and (c) is for a, b and c in Figure 1, respectively. Fold expression was calculated relative to untreated cells and experiments were repeated at least three times in triplicate. Data are presented as the mean ± SD; *$p < 0.05$, as compared with control (no treatment); $^{\#}p < 0.05$ for the indicated comparisons.

Supplementary Figure S3

a

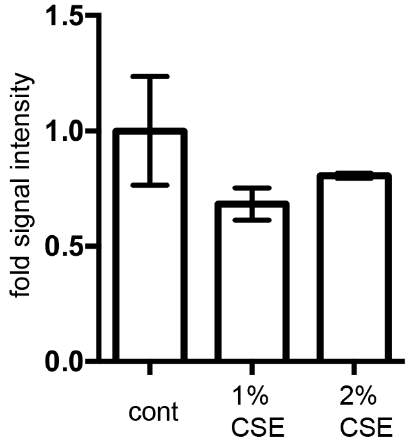

b

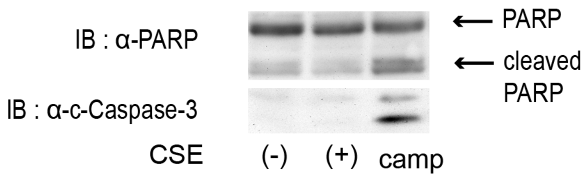

Supplementary Figure S3 | Effect of CSE on cell proliferation and cell death of A549 cells a. Cellular proliferation and its inhibition of A549 cells were determined by the CellTiter 96 Aqueous Non-Radioactive Cell Proliferation Assay™ (Promega). A549 cells were exposed to 1% or 2% CSE for 12h. Then, MTS (a tetrazolium compound) at 333 μg ml^{-1}+ phenazine methosulfate (at 25 μM) were added to each well of the 96-well plate for 1 h at 37°C. This allowed for the development of the reaction in which dehydrogenases reduce the MTS in metabolically active cells. Since the cells were not washed before the addition of MTS, we did not have any problem with potentially loosely adherent cells. The soluble MTS formazan product was measured at 490 nm with an autoreader (Micro-plate reader M-Tmax, Wako Ind., Ltd.). Optical density is directly proportional to the number of living cells in culture. Cytotoxicity (%) was calculated in the following way: [(OD of control cells −OD of drug-treated cells)/OD of control cells] × 100. b. A549 cells were exposed to 2% CSE or camptotesin (camp) for 12h. Then, the cell lysates underwent immunoblot using antibodies raised against PARP and caspase 3.

Supplementary Figure S4

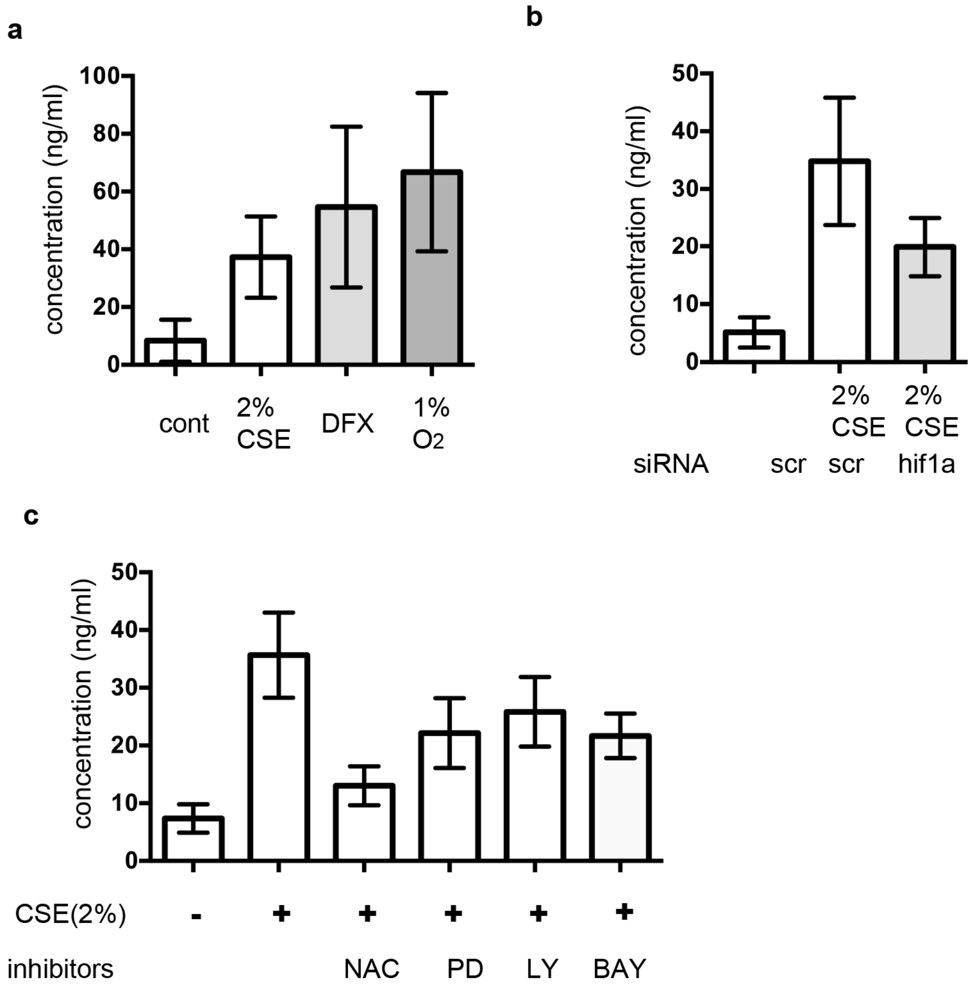

Supplementary Figure S4 | Results of VEGF ELISA

A549 ells were subjected to the indicated treatment for 6h and the culture supernatants were collected for VEGF assay (a, b and c). (b) A549 cells were transfected with the indicated small RNAs for 12 h and were subjected to the indicated treatment for 6h. (c) A549 ells were subjected to the indicated treatment for 6h with or without test reagents.

Supplementary Figure S5

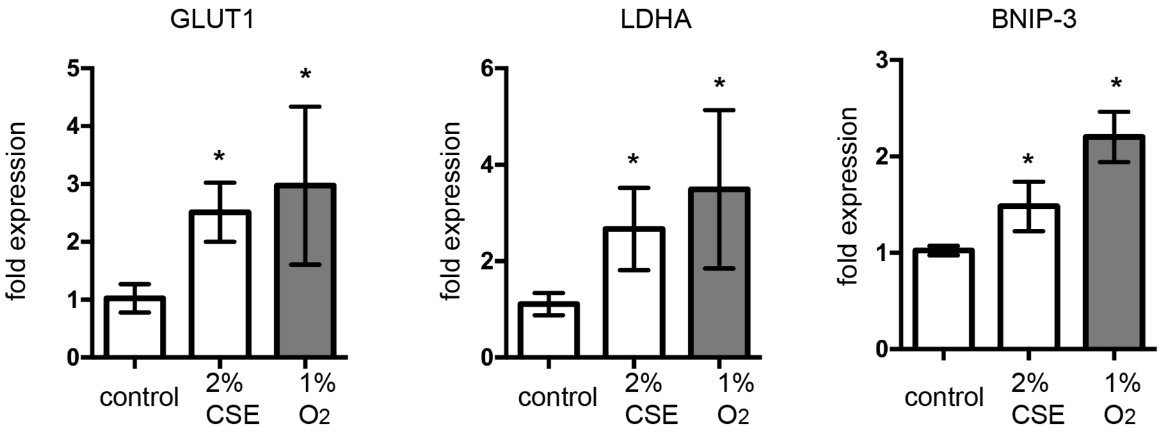

Supplementary Figure S5 | Impact of CSE on HIF-1-dependent gene expression A549 cells were culture for 6h with or without 2% CSE under 20% O_2 conditions and 1% O_2 conditions and harvested for semi-quantitative RT-PCR for glucose transporter 1 (GLUT1), lactate dehydrogenase A (LDHA), BCL2/adenovirus E1B 19 kd-interacting protein (BNIP-3). The fold expression was calculated on the value of CSE untreated cells. The fold activity was calculated on the value of CSE untreated cells. Experiments were repeated at least thrice in triplicate. Data are presented as the mean ± SD ;*$p < 0.05$ compared with the control (no treatment).

Supplementary Figure S6

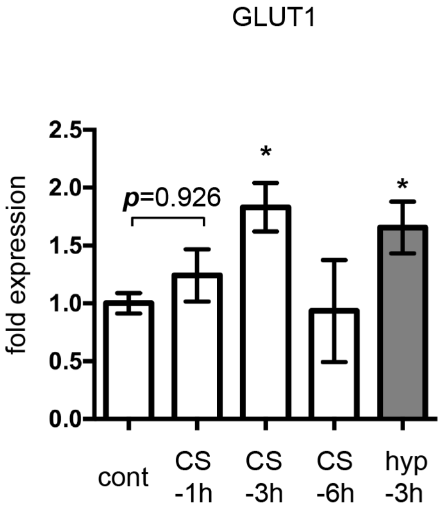

Supplementary Figure S6 | HIF-1 is activated in the lung in response to CS in mice Mice were divided into 5 groups: control (maintained in the air for 4h), CS-1h (exposed to CS for 50 m and maintained in the air for 1h), CS-3h (exposed to CS for 50 m and maintained in the air for 3h), CS-6h (exposed to CS for 50 m and maintained in the air for 6h) and hyp-3h (exposed to 1% O_2 for 50 m and maintained in the air for 3h). Mice were exposed to 10 filtered cigarettes using Smoke Generator in 50 minutes and mRNAs were harvested from lung tissue after incubation in the air for the indicated times. In the case of 1% O_2 conditions, mice were incubated in a chamber of which O_2 concentration was adjusted to 1%. Expression of GLUT1 was analyzed by semi-quantitative real-time RT-PCR using specific primers. Data are presented as the mean ± SD of three independent mice. *$p < 0.05$ compared with the control (20% and no treatment).
